# Zinc deficiency as a contributor to acute kidney injury in patients with chronic kidney disease: a propensity score–matched retrospective analysis

**DOI:** 10.3389/fnut.2025.1658308

**Published:** 2025-09-25

**Authors:** Yi-Chen Lai, Ting-Sian Yu, Kuo-Chuan Hung, Jheng-Yan Wu, Ping-Heng Tan, Chun-Ning Ho, I-Yin Hung, Chien-Ming Lin, I-Wen Chen

**Affiliations:** ^1^Department of Anesthesiology, Chi Mei Medical Center, Tainan City, Taiwan; ^2^School of Medicine, College of Medicine, National Sun Yat-sen University, Kaohsiung City, Taiwan; ^3^Department of Anesthesiology, E-Da Hospital, I-Shou University, Kaohsiung City, Taiwan; ^4^Department of Nutrition, Chi Mei Medical Center, Tainan City, Taiwan; ^5^Department of Anesthesiology, Chi Mei Medical Center, Liouying, Tainan City, Taiwan

**Keywords:** trace elements, kidney function decline, nutritional assessment, zinc level, risk factor

## Abstract

**Background:**

Acute kidney injury (AKI) is a frequent and serious complication in patients with chronic kidney disease (CKD) that often leads to poor clinical outcomes. Despite the biological plausibility of linking zinc deficiency (ZD) to increased AKI susceptibility, large-scale evidence evaluating this association in the CKD population remains scarce.

**Methods:**

This retrospective cohort study utilized the TriNetX Analytics Network Platform to identify patients aged ≥18 years with pre-existing CKD who underwent serum zinc testing between January 2010 and December 2023. Patients were categorized into zinc deficiency (ZD: <70 μg/dl) and control groups (70–120 μg/dl). After applying the exclusion criteria and 1:1 propensity score matching for demographics, comorbidities, laboratory parameters, and medications, we analyzed 5,619 patients per group. The primary outcome was new-onset AKI at the 12-month follow-up, with secondary outcomes including risk of mortality, end-stage renal disease (ESRD), intensive care unit (ICU) admission, and major cardiac adverse events (MCAEs).

**Results:**

At 12 months, zinc-deficient patients experienced significantly higher risks of AKI [19.3 vs. 14.9%; hazard ratio (HR): 1.37, 95% confidence interval (CI): 1.25–1.50, *P* < 0.001], mortality (9.0 vs. 4.8%; HR 1.95, 95% CI 1.68–2.26, *P* < 0.001), ESRD progression (1.9 vs. 1.4%; HR 1.40, 95% CI 1.04–1.88, *P* = 0.025), and ICU admissions (8.7 vs. 5.8%; HR 1.56, 95% CI 1.35–1.79, *P* < 0.001). The effects were stronger at 6 months than at 12 months, suggesting rapid manifestation. Notably, these zinc-specific effects persisted even after excluding patients who developed malnutrition, reinforcing that the observed associations were attributable to zinc deficiency rather than general nutritional status. Subgroup analyses demonstrated consistent findings across diverse patient characteristics.

**Conclusions:**

Baseline zinc deficiency is an independent, modifiable risk factor for AKI development and mortality in patients with CKD. These findings support routine zinc status assessments and targeted supplementation strategies in CKD management protocols. However, the observational design and single-point zinc measurements limit the causal inferences. Prospective randomized controlled trials are needed to determine whether zinc supplementation can prevent AKI and improve outcomes in patients with CKD.

## 1 Introduction

Acute kidney injury (AKI) is one of the most serious complications in clinical medicine, affecting approximately 20% of hospitalized patients worldwide, with mortality rates exceeding 50% in critically ill populations (1–3). This sudden deterioration in kidney function threatens immediate survival and establishes long-term consequences, including accelerated chronic kidney disease (CKD) progression, increased cardiovascular morbidity, and substantially elevated healthcare costs that persist for decades (4–8). Traditional risk factors for AKI include advanced age, pre-existing CKD, diabetes mellitus, cardiovascular disease, sepsis, major surgery, and nephrotoxic medication exposure (1, 9–13). However, these established factors primarily represent non-modifiable patient characteristics or unavoidable clinical scenarios, limiting the opportunities for preventive intervention. AKI prevention has therefore emerged as a critical clinical priority, particularly given the limited therapeutic options available once kidney injury occurs (14, 15). This therapeutic limitation underscores the importance of primary prevention by identifying modifiable risk factors before kidney injury develops.

Zinc plays fundamental roles in immune function, wound healing, and cellular repair mechanisms (16–19). Beyond general malnutrition, zinc deficiency has unique pathophysiological relevance to AKI. It impairs antioxidant defenses, disrupts immune regulation, and compromises cellular repair, thereby heightening renal vulnerability to ischemic or toxic insults. Patients with CKD commonly develop zinc deficiency due to decreased dietary intake, impaired absorption, increased urinary losses, and chronic inflammation that characterizes progressive kidney disease (20–22). At the cellular level, zinc deficiency compromises antioxidant defense systems, impairs immune responses, and disrupts normal tissue repair processes (23). These physiological disruptions create a concerning scenario in which zinc-deficient patients with CKD may face increased susceptibility to infections, delayed recovery from acute illness, and accelerated progression of kidney damage (20, 24–26). Experimental studies also support the notion that zinc deficiency may directly worsen kidney injury, showing that zinc supplementation reduces diabetic renal damage by inducing metallothionein and suppressing oxidative stress and fibrosis-related pathways (27). In ischemia-reperfusion models, zinc improves antioxidant defenses and partially preserves kidney function (28) while also activating protective hypoxia-inducible factors in a dose-dependent manner (29). These mechanisms suggest zinc deficiency may act as a direct contributor to AKI risk rather than solely reflecting poor nutritional status.

Recent studies have shown that lower dietary zinc intake is associated with a higher risk of developing CKD (30, 31). In critically ill patients with AKI, zinc supplementation has been associated with improved survival, particularly in those with early stage AKI and sepsis (32). Few epidemiologic studies have disentangled the specific contribution of zinc deficiency from other nutritional and metabolic risk factors for AKI, particularly in patients with CKD. Large-scale human data are needed to clarify whether zinc deficiency independently predisposes to AKI and to inform potential interventional strategies. To address this gap, the present study aimed to evaluate the association between baseline zinc deficiency and the risk of AKI and renal function decline in patients with pre-existing CKD using a large, real-world healthcare database.

## 2 Methods

### 2.1 Data sources

This retrospective cohort study utilized data from the TriNetX Analytics Network Platform, a federated health research network that aggregates electronic health records from healthcare organizations worldwide. The TriNetX platform provides access to de-identified patient data from over 140 healthcare organizations, with the majority of participating institutions located in the United States. The dataset encompasses comprehensive clinical information, including demographic characteristics, laboratory results, diagnostic codes, medication prescriptions, and procedural records, enabling robust epidemiological research across diverse patient populations. The TriNetX database has been widely employed in peer-reviewed clinical and epidemiological studies, supporting its reliability and validity as a research resource (33–35). The study protocol was approved by the Institutional Review Board of Chi Mei Medical Center (IRB number: 11310-E04), which granted a waiver of informed consent due to the retrospective nature of the study design and the use of de-identified data that posed minimal risk to patient privacy. This waiver aligns with the standard ethical guidelines for observational research utilizing pre-existing electronic health records without direct patient interaction or intervention.

### 2.2 Study population and eligibility criteria

We identified eligible participants by selecting patients aged 18 years and older who underwent serum zinc level testing between January 1, 2010, and December 31, 2023. This broad timeframe was chosen to capture sufficient sample sizes while ensuring adequate follow-up periods for outcome assessment. Patients were categorized into two primary groups based on their serum zinc concentrations: the zinc deficiency group (ZD group) included patients with serum zinc levels below 70 μg/dl, while the control group comprised patients with serum zinc levels between 70 and 120 μg/dl. Zinc deficiency was defined as a serum zinc concentration <70 μg/dl, consistent with established clinical laboratory reference ranges and prior studies evaluating zinc status in chronic disease populations (36). Information regarding the specific assay methods used for serum zinc measurement across participating institutions in the TriNetX network is not available; however, all results are harmonized and reported in standardized units (μg/dl).

The date of zinc testing served as the index date for all subsequent analyses, providing a clear temporal reference point for outcome measurements and follow-up calculations. All eligible patients were required to have an established diagnosis of CKD before the index date, ensuring that we were studying patients with pre-existing renal dysfunction rather than those developing kidney disease *de novo*.

### 2.3 Exclusion criteria

To ensure the validity of our findings and minimize confounding by acute medical conditions, we implemented several exclusion criteria. Patients with a documented history of CKD stage 5 or end-stage renal disease (ESRD) before the index date were excluded, as these individuals represent a distinct population with irreversible kidney dysfunction. Additionally, we excluded patients who experienced sepsis, pneumonia, intensive care unit (ICU) admission, AKI, or COVID-19 infection within 1 month prior to the index date. These exclusions were made because acute illness can significantly affect zinc metabolism, serum zinc levels, and kidney function, potentially confounding the relationship between the baseline zinc status and subsequent renal outcomes.

### 2.4 Data collection and matching strategy

Baseline characteristics and comorbidities were extracted from the 3-year period preceding the index date to capture patient health status. To ensure a balanced comparison of the groups, we implemented a 1:1 propensity score-matching approach. The matching algorithm incorporated essential demographic variables, including age, sex, race, and body mass index (BMI), as well as laboratory parameters, such as estimated glomerular filtration rate (eGFR), serum albumin, hemoglobin A1c (HbA1c), and hemoglobin levels.

To minimize confounding by CKD severity, we additionally matched the patients for the presence of specific CKD stages, ensuring that both groups had similar distributions of baseline kidney function. Furthermore, we adjusted for the use of medications with established renoprotective effects, including second-line antidiabetic agents such as SGLT2 inhibitors and antihypertensive therapies such as ACE inhibitors and ARBs, as these treatments could potentially confound the association between zinc status and kidney disease progression. To further address potential treatment bias, we specifically matched for zinc supplementation. Patients receiving parenteral nutrition or with liver disease were not excluded *a priori*. Instead, serum albumin, malnutrition and liver disease status were included as covariates in the propensity score matching to reduce potential confounding related to zinc metabolism.

### 2.5 Study outcomes

The primary outcome was new-onset AKI at 12-month follow-up. AKI was identified using International Classification of Diseases (ICD) diagnostic codes (N17) recorded in the TriNetX database, consistent with common epidemiological approaches in large-scale research. This endpoint was selected because AKI represents critical acute deterioration in kidney function that can lead to permanent damage, particularly in patients with pre-existing CKD. Secondary outcomes included the risks of all-cause mortality, ESRD, ICU admission, and major cardiac adverse events (MCAEs) encompassing cardiac arrest, ventricular arrhythmias, atrial fibrillation and flutter, and myocardial infarction.

### 2.6 Subgroup analyses

To examine whether the effect of zinc deficiency varied across different patient profiles, we conducted pre-specified subgroup analyses based on key clinical characteristics. These included age (18–50 vs. >50 years), sex, hypertension status, duration of diabetes, presence of obesity, and anemia. This approach allowed us to assess potential effect modifiers and identify subpopulations that may be more vulnerable to zinc deficiency-related complications.

### 2.7 Sensitivity analysis

Recognizing that zinc deficiency frequently occurs alongside malnutrition, which could independently contribute to adverse renal outcomes (37), we conducted a sensitivity analysis to test the robustness of our findings. In this analysis, we excluded patients who developed malnutrition during the 12-month follow-up period and re-examined whether zinc deficiency remained a significant risk factor for AKI and CKD progression. This approach allowed us to determine whether the observed associations were specifically attributable to zinc deficiency or could be explained by concurrent nutritional deficiencies.

### 2.8 Statistical analysis

Continuous variables are presented as means with standard deviations, while categorical variables are expressed as frequencies and percentages. To achieve an optimal balance between the zinc deficiency and control groups, we implemented propensity score matching using a greedy nearest-neighbor algorithm with appropriate caliper settings. The quality of matching was assessed using standardized mean differences (SMD), with values less than 0.1 indicating adequate balance between groups, supplemented by visual inspection of propensity score distributions to ensure appropriate overlap.

Time-to-event outcomes were analyzed using Kaplan–Meier survival curves, with between-group differences assessed using the log-rank test. Cox proportional hazards regression models were employed to calculate hazard ratios (HRs) with 95% confidence intervals (CIs), allowing for the appropriate handling of censored data and time-varying risk. For subgroup analyses, the statistical significance of the differences between subgroups was evaluated by examining the confidence interval overlap, a conservative approach that reduces the likelihood of false-positive findings while maintaining clinical interpretability.

Multivariate Cox regression analysis was performed to identify independent predictors of AKI development, incorporating all clinically relevant covariates that achieved a balance through the matching process. All statistical analyses were conducted using the TriNetX platform's integrated analytical tools, with a two-sided *P*-value threshold of less than 0.05 considered statistically significant throughout all analyses.

## 3 Results

### 3.1 Patient selection and baseline characteristics before and after matching

**Figure 1:**
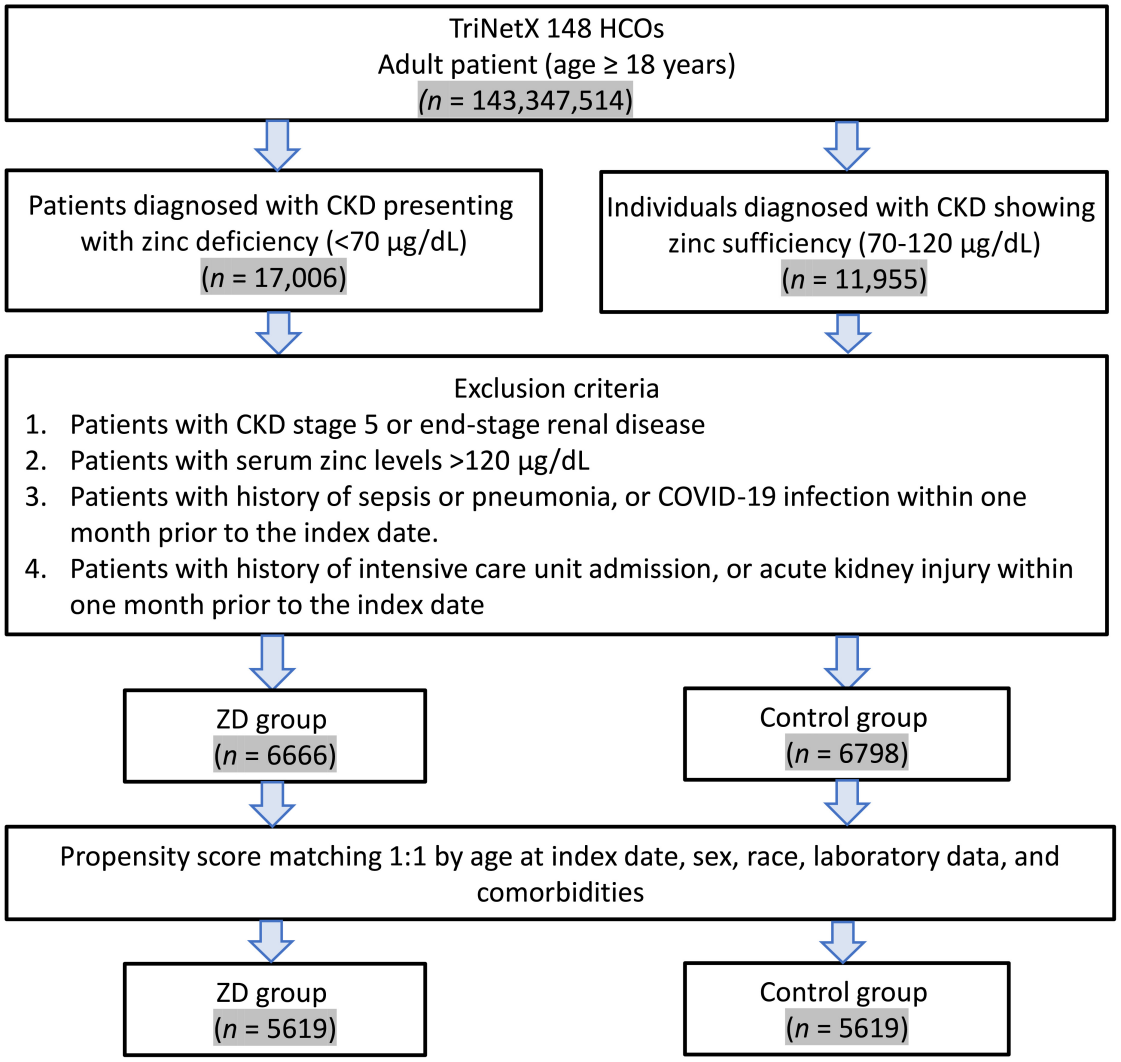
Patient selection flowchart from the TriNetX database. The flowchart illustrates the systematic exclusion process applied to identify eligible patients with zinc deficiency (ZD) and zinc sufficiency (control group). HCOs, Healthcare Organizations.

The patient selection process is illustrated in Figure 1. From the TriNetX database, we initially identified 28,961 eligible patients with a previous history of CKD who underwent serum zinc level testing between January 1, 2010, and December 31, 2023. After applying the exclusion criteria, 6,666 patients remained in the zinc deficiency group and 6,798 in the control group, respectively. Before propensity score matching, significant baseline differences existed between the two groups, as demonstrated by standardized mean differences (SMD) greater than 0.1 for several variables (Table 1). The zinc deficiency group exhibited a higher prevalence of anemia (47.1 vs. 38.7%, SMD = 0.170), heart failure (28.2 vs. 22.6%, SMD = 0.128), malnutrition (16.8 vs. 12.2%, SMD = 0.130), and alcohol-related disorders (6.9 vs. 4.6%, SMD = 0.102). Additionally, the zinc deficiency group demonstrated lower serum albumin levels, with 84.9% having albumin ≥3.5 g/dl compared to 89.8% in the control group (SMD = 0.149).

**Table 1:** Baseline characteristics of patients before and after propensity score matching.

**Variables**	**Before matching**			**After matching**		
	**ZD group (*****n*** = **6,666)**	**Control group (*****n*** = **6,798)**	**SMD** ^†^	**ZD group (*****n*** = **5,619)**	**Control group (*****n*** = **5,619)**	**SMD** ^†^
**Patient characteristics**						
Age at index (years)	64.8 ± 15.5	63.1 ± 15.5	0.104	64.0 ± 15.7	63.8 ± 15.4	0.007
Female	3,699 (55.5%)	3,854 (56.7%)	0.024	3,180 (56.6%)	3,166 (56.3%)	0.005
BMI kg/m^2^	31.0 ± 9.7	31.7 ± 9.2	0.068	31.4 ± 9.6	31.5 ± 9.3	0.014
White	4,445 (66.7%)	4,539 (66.8%)	0.002	3,740 (66.6%)	3,723 (66.3%)	0.006
**Comorbidities**						
Essential (primary) hypertension	4,954 (74.3%)	5,061 (74.4%)	0.003	4,185 (74.5%)	4,176 (74.3%)	0.004
Disorders of lipoprotein metabolism and other lipidemias	4,159 (62.4%)	4,540 (66.8%)	0.092	3,647 (64.9%)	3,628 (64.6%)	0.007
**CKD**						
Stage 1	235 (3.5%)	278 (4.1%)	0.029	210 (3.7%)	218 (3.9%)	0.007
Stage 2	972 (14.6%)	1,127 (16.6%)	0.055	885 (15.8%)	853 (15.2%)	0.016
Stage 3	3,484 (52.3%)	3,573 (52.6%)	0.006	2,948 (52.5%)	2,937 (52.3%)	0.004
Stage 4	641 (9.6%)	472 (6.9%)	0.097	460 (8.2%)	440 (7.8%)	0.013
Diabetes mellitus	3,084 (46.3%)	3,143 (46.2%)	0.001	2,614 (46.5%)	2,605 (46.4%)	0.003
Neoplasms	3,002 (45.0%)	3,098 (45.6%)	0.011	2,558 (45.5%)	2,550 (45.4%)	0.003
Overweight and obesity	2,826 (42.4%)	3,118 (45.9%)	0.070	2,520 (44.8%)	2,502 (44.5%)	0.006
Anemias	3,138 (47.1%)	2,631 (38.7%)	0.170	2,459 (43.8%)	2,450 (43.6%)	0.003
Vitamin D deficiency	2,249 (33.7%)	2,490 (36.6%)	0.061	1,973 (35.1%)	1,967 (35.0%)	0.002
Chronic lower respiratory diseases	2,247 (33.7%)	2,245 (33.0%)	0.015	1,893 (33.7%)	1,881 (33.5%)	0.005
Ischemic heart diseases	2,343 (35.1%)	2,154 (31.7%)	0.073	1,887 (33.6%)	1,894 (33.7%)	0.003
Heart failure	1,879 (28.2%)	1,538 (22.6%)	0.128	1,450 (25.8%)	1,412 (25.1%)	0.016
Diseases of liver	1,630 (24.5%)	1,533 (22.6%)	0.045	1,311 (23.3%)	1,308 (23.3%)	0.001
Other cardiac arrhythmias	1,407 (21.1%)	1,223 (18.0%)	0.079	1,091 (19.4%)	1,090 (19.4%)	<0.001
Atrial fibrillation and flutter	1,306 (19.6%)	1,090 (16.0%)	0.093	1,013 (18.0%)	988 (17.6%)	0.012
Cerebrovascular diseases	1,249 (18.7%)	1,089 (16.0%)	0.072	980 (17.4%)	962 (17.1%)	0.008
Malnutrition	1,119 (16.8%)	831 (12.2%)	0.130	770 (13.7%)	794 (14.1%)	0.012
Nicotine dependence	877 (13.2%)	777 (11.4%)	0.053	691 (12.3%)	677 (12.0%)	0.008
COVID-19	496 (7.4%)	536 (7.9%)	0.017	433 (7.7%)	433 (7.7%)	<0.001
Alcohol related disorders	463 (6.9%)	311 (4.6%)	0.102	295 (5.3%)	296 (5.3%)	0.001
**Laboratory data**						
Hemoglobin ≥12 mg/dl	4,970 (74.6%)	5,574 (82.0%)	0.181	4,452 (79.2%)	4,444 (79.1%)	0.004
Hemoglobin A1c ≥7%	1,555 (23.3%)	1,681 (24.7%)	0.033	1,362 (24.2%)	1,361 (24.2%)	<0.001
Albumin g/dl (≥3.5 g/dl)	5,659 (84.9%)	6,106 (89.8%)	0.149	4,986 (88.7%)	4,955 (88.2%)	0.017
eGFR >60 ml/min/1.73 m^2^	4,277 (64.2%)	4,501 (66.2%)	0.043	3,689 (65.7%)	3,660 (65.1%)	0.011
**Medications**						
Antilipemic agents	3,607 (54.1%)	3,795 (55.8%)	0.034	3,107 (55.3%)	3,081 (54.8%)	0.009
Insulins and analogs	2,412 (36.2%)	2,254 (33.2%)	0.064	1,975 (35.1%)	1,973 (35.1%)	0.001
ACE inhibitors	1,952 (29.3%)	1,957 (28.8%)	0.011	1,660 (29.5%)	1,647 (29.3%)	0.005
Angiotensin II inhibitor	1,793 (26.9%)	1,933 (28.4%)	0.034	1,570 (27.9%)	1,573 (28.0%)	0.001
GLP-1 analogs	545 (8.2%)	657 (9.7%)	0.052	504 (9.0%)	488 (8.7%)	0.010
Zinc supplementation	483 (7.2%)	581 (8.5%)	0.048	452 (8.0%)	469 (8.3%)	0.011
SGLT2 inhibitors	370 (5.6%)	416 (6.1%)	0.024	327 (5.8%)	320 (5.7%)	0.005

BMI, body mass index; SMD, standardized mean differences; eGFR, estimated Glomerular Filtration Rate; COPD, chronic obstructive pulmonary disease; GLP-1, Glucagon-like peptide-1; SGLT2, Sodium-glucose co-transporter 2; CKD, Chronic kidney disease.

^†^SMD values <0.1 indicate adequate balance between groups.

**Figure 2:**
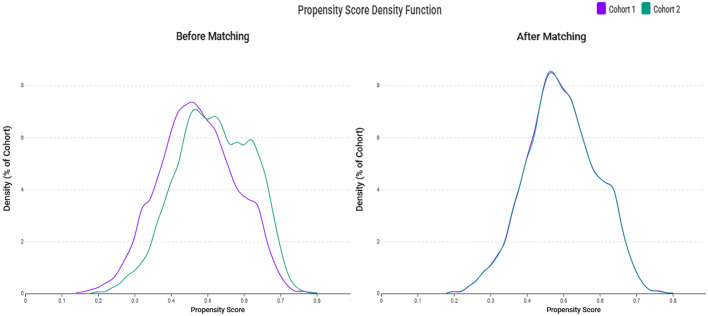
Propensity score density distributions before and after matching. The left panel shows imbalanced distributions between the zinc deficiency (Cohort 1) and control groups (Cohort 2) before matching. The right panel demonstrates improved alignment and covariate balance after 1:1 matching with a 0.1 standard deviation caliper.

Following matching, we successfully matched 5,619 patients in each group. The matching process achieved excellent balance between groups, as evidenced by all SMD values falling below 0.1 (Table 1). Figure 2 demonstrates the improved overlap and balance in the propensity score distributions after matching. The matched cohorts showed similar demographic characteristics, with a mean age of 64.0 ± 15.7 years in the zinc deficiency group and 63.8 ± 15.4 years in the control group. Female patients comprised 56.6 and 56.3% of patients in the zinc deficiency and control groups, respectively. The prevalence of major comorbidities, including CKD stage 3 (52.5 vs. 52.3%), diabetes mellitus (46.5 vs. 46.4%), and essential hypertension (74.5 vs. 74.3%), was well balanced between groups.

### 3.2 Association of zinc deficiency and 12-month outcomes

At the 12-month follow-up, AKI occurred in nearly one in five zinc-deficient patients (1,087 patients, 19.3%) compared to fewer than one in seven controls (837 patients, 14.9%), representing a 37% increase in risk (HR: 1.37, 95% CI: 1.25–1.50, *P* < 0.001; Table 2). Beyond acute deterioration, zinc deficiency also accelerates progression toward irreversible kidney damage. Although ESRD remained relatively uncommon in both groups, zinc-deficient patients faced a 40% higher risk of progressing to this outcome (HR: 1.40, 95% CI: 1.04–1.88, *P* = 0.025), indicating that zinc deficiency plays a role not only in precipitating AKI but also in accelerating the progression toward irreversible kidney failure.

**Table 2:** Association between zinc deficiency and 12-month outcomes.

**Outcomes**	**ZD group (*n* = 5,619)**	**Control group (*n* = 5,619)**	**HR (95% CI)**	***P*-value**
	**Events (%)**	**Events (%)**		
AKI	1,087 (19.3%)	837 (14.9%)	1.37 (1.25–1.50)	<0.001
ESRD	104 (1.9%)	77 (1.4%)	1.40 (1.04–1.88)	0.025
Mortality	503 (9.0%)	267 (4.8%)	1.95 (1.68–2.26)	<0.001
ICU	488 (8.7%)	326 (5.8%)	1.56 (1.35–1.79)	<0.001
MCAEs	1,406 (25.0%)	1,318 (23.5%)	1.10 (1.02–1.19)	0.012

AKI, acute kidney injury; ESRD, end-stage renal disease; ICU, intensive care units; MCAE, major cardiac adverse events; ZD, zinc deficiency; HR, hazard ratio; CI, confidence interval.

In addition, zinc-deficient patients experienced nearly double the mortality rate (9.0%) compared to controls (4.8%; HR: 1.95, 95% CI: 1.68–2.26, *P* < 0.001). Intriguingly, this dramatic mortality increase could not be explained by cardiovascular events alone, as cardiac complications showed only a mild elevation (25.0% vs. 23.5%, HR: 1.10, 95% CI: 1.02–1.19, *P* = 0.012). These findings imply that zinc deficiency exerts its fatal influence through multiple potentially non-cardiovascular pathways in this patient population. Supporting this broader vulnerability, ICU admissions occurred 56% more frequently in zinc-deficient patients (8.7%) than in controls (5.8%; HR: 1.56, 95% CI: 1.35–1.79, *P* < 0.001), indicating that zinc deficiency not only increases the risk of severe complications but also substantially elevates healthcare resource utilization.

### 3.3 Association of zinc deficiency and 6-month outcomes

To understand how quickly these adverse effects manifest, we examined the outcomes at the 6-month follow-up (Table 3). The results revealed that the effects of zinc deficiency were stronger at 6 months than at 12 months. The AKI risk increased by 49% at 6 months (13.3% vs. 9.3%, HR: 1.49) compared to 37% at 12 months, suggesting that zinc deficiency creates immediate physiological stress rather than a gradual decline. The 65% increase in ESRD progression at 6 months (vs. 40% at 12 months) likely reflects that the most vulnerable patients progressed rapidly to end-stage disease early on.

**Table 3:** Association between zinc deficiency and 6-month outcomes.

**Outcomes**	**ZD group (*n* = 5,619)**	**Control group (*n* = 5,619)**	**HR (95% CI)**	***P*-value**
	**Events (%)**	**Events (%)**		
AKI	749 (13.3%)	523 (9.3%)	1.49 (1.33–1.66)	<0.001
ESRD	63 (1.1%)	39 (0.7%)	1.65 (1.10–2.45)	0.014
Mortality	322 (5.7%)	174 (3.1%)	1.89 (1.57–2.27)	<0.001
ICU	329 (5.9%)	208 (3.7%)	1.62 (1.36–1.93)	<0.001
MCAEs	1,141 (20.3%)	1,061 (18.9%)	1.10 (1.02–1.20)	0.020

AKI, acute kidney injury; ESRD, end-stage renal disease; ICU, intensive care units; MCAE, major cardiac adverse events; ZD, zinc deficiency; HR, hazard ratio; CI, confidence interval.

Remarkably, mortality and ICU admission risks remained virtually identical at both time points —mortality nearly doubled (HR: 1.89 at 6 months, 1.95 at 12 months) and ICU admissions increased by approximately 60% at both intervals. This consistent pattern of adverse outcomes suggests that zinc deficiency induces widespread physiological vulnerability, impacting multiple organ systems concurrently. These findings suggest a critical early window when the effects of zinc deficiency are most pronounced, emphasizing the clinical importance of prompt recognition and correction.

### 3.4 Sensitivity analysis: association of baseline zinc deficiency and 12-month outcomes after excluding patients with malnutrition

To assess whether zinc deficiency causes direct harm or simply reflects general malnutrition, we conducted a sensitivity analysis excluding patients who developed malnutrition during follow-up, leaving 4,863 patients in each group for this “cleaner” comparison (Table 4). The results revealed that AKI, mortality, and ICU admissions remained significantly elevated, even after the removal of malnourished patients. This persistence implies that zinc deficiency exerts direct, independent biological effects on kidney function and survival through zinc-specific pathways.

**Table 4:** Sensitivity analysis of association between zinc deficiency and 1-year outcomes after excluding patients with malnutrition.

**Outcomes**	**ZD group (*n* = 4,863)**	**Control group (*n* = 4,863)**	**HR (95% CI)**	***P*-value**
	**Events (%)**	**Events (%)**		
AKI	760 (15.6%)	598 (12.3%)	1.32 (1.19–1.47)	<0.001
ESRD	74 (1.5%)	57 (1.2%)	1.33 (0.94–1.88)	0.102
Mortality	328 (6.7%)	183 (3.8%)	1.84 (1.53–2.20)	<0.001
ICU	276 (5.7%)	213 (4.4%)	1.33 (1.11–1.59)	0.002
MCAEs	1,100 (22.6%)	1,103 (22.7%)	1.02 (0.93–1.10)	0.726

AKI, acute kidney injury; ESRD, end-stage renal disease; ICU, intensive care units; MCAE, major cardiac adverse events; ZD, zinc deficiency; HR, hazard ratio; CI, confidence interval.

However, the associations with cardiac complications (HR, 1.02; *P* = 0.726) and ESRD (HR, 1.33; *P* = 0.102) were no longer statistically significant. This attenuation suggests that zinc deficiency may exert its influence through two distinct pathways: one involving direct, zinc-specific biological mechanisms that precipitate acute complications and increase mortality risk and another involving indirect effects mediated by broader nutritional deficits, which may play a greater role in long-term cardiovascular outcomes and CKD progression.

### 3.5 Subgroup analysis

**Figure 3:**
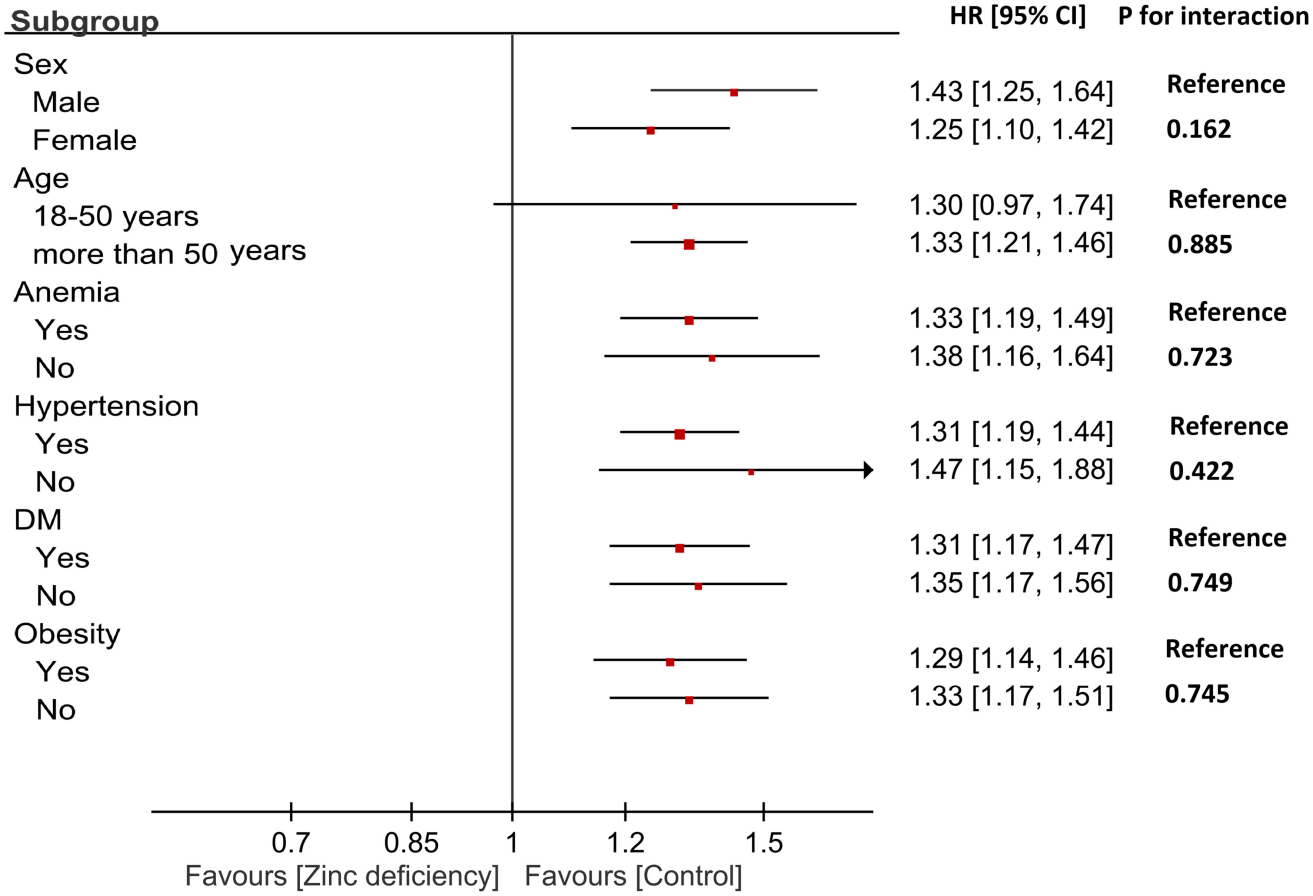
Subgroup analysis of the association between zinc deficiency and risk of acute kidney injury (AKI). Hazard ratios (HRs) and 95% confidence intervals are shown for each subgroup. The interaction *P*-values indicate no significant effect modification across sex, age, anemia, hypertension, diabetes mellitus (DM), or obesity status.

Subgroup analyses revealed remarkably consistent associations between zinc deficiency and AKI across all patient characteristics (Figure 3). Male patients showed a slightly stronger association (HR: 1.43) than females (HR: 1.25), while age groups demonstrated similar risks (HR: 1.30 for ages 18–50; HR: 1.33 for >50 years), though these differences were not statistically significant. Importantly, comorbid conditions did not modify the impact of zinc deficiency. These findings suggest that zinc deficiency represents a fundamental and independent risk factor for AKI, making it equally important across diverse patient populations with CKD.

### 3.6 Risk factors for AKI

Multivariate Cox regression analysis identified zinc deficiency as an independent predictor of new-onset AKI (Table 5). After adjusting for demographic characteristics and relevant comorbidities, zinc deficiency remained significantly associated with AKI development (HR 1.44, 95% CI: 1.32–1.56, *P* < 0.001). Several other factors have emerged as significant independent predictors of AKI. Malnutrition demonstrated the strongest association (HR 1.81, 95% CI: 1.64–2.00, *P* < 0.001), followed by CKD (HR 1.70, 95% CI: 1.46–1.99, *P* < 0.001) and anemia (HR 1.57, 95% CI: 1.44–1.72, *P* < 0.001). Heart failure was also a significant predictor (HR 1.51, 95% CI: 1.38–1.66, *P* < 0.001), along with liver diseases (HR 1.29, 95% CI: 1.18–1.42, *P* < 0.001).

**Table 5:** Risk factors for new-onset acute kidney injury at 12-month follow-up.

**Variable**	**HR (95% CI)^†^**	***P*-value**
ZD vs. control groups	1.44 (1.32, 1.56)	<0.001
Male	1.18 (1.08, 1.28)	<0.001
Age at Index	1.00 (1.00, 1.00)	0.867
Chronic kidney disease (CKD)	1.70 (1.46, 1.99)	<0.001
Diabetes mellitus	1.18 (1.09, 1.29)	<0.001
Malnutrition	1.81 (1.64, 2.00)	<0.001
Ischemic heart diseases	1.23 (1.12, 1.35)	<0.001
Neoplasms	1.13 (1.04, 1.23)	0.005
Diseases of liver	1.29 (1.18, 1.42)	<0.001
Heart failure	1.51 (1.38, 1.66)	<0.001
Anemias	1.57 (1.44, 1.72)	<0.001
Essential (primary) hypertension	1.15 (1.03, 1.29)	0.011
Overweight and obesity	0.92 (0.84, 1.00)	0.056

HR, hazard ratio; CI, confidence interval.

^†^Multivariate logistic regression analysis.

## 4 Discussion

Our investigation revealed that zinc-deficient CKD patients face substantially elevated risks across multiple clinical endpoints, with the most pronounced effects observed for mortality and AKI. Temporal analysis indicates that these adverse effects manifest rapidly, with stronger associations evident at 6 months compared to 12 months, suggesting that zinc deficiency creates immediate physiological vulnerability rather than gradual deterioration. Importantly, our sensitivity analysis, excluding patients who developed malnutrition, confirmed that zinc deficiency exerts direct, independent biological effects on kidney function and survival, distinguishing it from general nutritional inadequacy. The consistency of findings across diverse patient subgroups underscores the fundamental importance of adequate Zn status in maintaining kidney health and overall clinical stability in this vulnerable population.

The association between zinc deficiency and increased AKI risk aligns with emerging evidence highlighting the critical role of zinc in kidney health, although our study provides the most comprehensive epidemiological evidence to date. While previous studies have examined the impact of zinc intake on chronic renal function decline (30, 31) and investigated outcomes in patients with AKI (32), few studies have systematically evaluated whether baseline zinc status predicts the development of AKI in the broader CKD population. Our findings build on prior research by showing that baseline zinc deficiency serves as an independent predictor of AKI in diverse clinical scenarios. The biological plausibility of our findings rests on the fundamental roles of zinc in cellular repair mechanisms and antioxidant defense systems (17–19). Zinc serves as a cofactor for numerous enzymes involved in DNA repair, protein synthesis, and maintenance of cellular membrane integrity, processes that are particularly crucial during AKI when tubular epithelial cells face oxidative stress and require rapid regeneration. Additionally, zinc deficiency compromises immune function, potentially increasing susceptibility to infections that can precipitate episodes (24). The stronger association observed at 6 months suggests that zinc deficiency creates an immediate state of physiological vulnerability, making kidneys less resilient to various insults, including nephrotoxic medications, hemodynamic changes, and inflammatory processes.

Our findings demonstrate that baseline zinc deficiency is associated with an increased risk of progression to ESRD in patients with CKD, which is consistent with the results of Tokuyama et al. (38), who identified low serum zinc as an independent predictor of ESRD. Their study (38) also identified an interaction between zinc status and hypoalbuminemia, suggesting that zinc deficiency may exert its nephrotoxic effects more strongly in malnourished patients. Consistent with Tokuyama et al. (38), our results confirm that zinc deficiency predicts ESRD; however, our study extends these findings in several important ways. First, our study included a substantially larger and more diverse population drawn from a multinational real-world dataset, thereby improving generalizability. Second, unlike the previous study, we excluded patients with advanced renal failure at baseline and incorporated propensity score matching to account for comorbidities, nutritional status, and concurrent medications including zinc supplementation. Third, our sensitivity analysis, excluding patients who developed malnutrition during follow-up, revealed that the association between zinc deficiency and ESRD was attenuated and no longer statistically significant. This suggests that the impact of zinc on long-term renal decline may, in part, be mediated through broader nutritional mechanisms, a nuance that was not explored in previous work (38). However, we recognize that this interval may underestimate longer-term outcomes such as ESRD progression, given the typically slower trajectory of CKD to ESRD. Our results highlight the need for future prospective trials to determine whether zinc repletion alters the trajectory of CKD progression, particularly in nutritionally vulnerable subgroups.

Our large-scale analysis provides epidemiological evidence for the relationship between zinc status and survival in populations with CKD. Zinc deficiency is correctable, making it a vital and often overlooked target for improving survival. A key advance of this study is the finding that zinc deficiency independently predicts not only AKI but also all-cause mortality (HR = 1.95) and ICU admission (HR = 1.56) in patients with CKD. Importantly, these associations persisted even after excluding patients with malnutrition, suggesting that zinc deficiency confers additional risk beyond general nutritional status. The observation that MCAEs alone cannot explain the increase in mortality suggests that zinc deficiency affects multiple organ systems simultaneously, potentially through its effects on immune function, wound healing, and cellular repair mechanisms that are critical for recovery from acute illness.

The mild elevation in MCAEs associated with zinc deficiency provides important insights into the cardiovascular effects of micronutrient deficiencies in patients with CKD. Current evidence suggests that zinc deficiency can impair endothelial function, promote oxidative stress, and disrupt normal cardiac rhythm regulation (39–41). The attenuation of associations between MACEs and zinc deficiency in our sensitivity analysis excluding malnourished patients suggests that zinc deficiency may influence cardiovascular outcomes partially through pathways involving broader nutritional status. In contrast, the persistence of elevated mortality and ICU admission risks supports the hypothesis that zinc deficiency may primarily drive non-cardiovascular mortality, potentially through mechanisms such as impaired immune function or increased susceptibility to infection and critical illness. Further studies are warranted to clarify the predominant pathways by which zinc deficiency impacts adverse outcomes in CKD. The relatively mild cardiac risk elevation compared to other outcomes may reflect the multifactorial nature of cardiovascular disease in patients with CKD (42), where zinc deficiency represents one of many contributing factors.

The remarkable consistency of the association between zinc deficiency and AKI across all examined patient subgroups strengthens the evidence for a fundamental biological relationship. The absence of significant effect modification by age, sex, diabetes status, or other major comorbidities suggests that zinc deficiency is a universal risk factor that operates independently of traditional clinical characteristics. This finding has important clinical implications, indicating that zinc status assessment and optimization should be considered across diverse CKD populations rather than being restricted to specific high-risk subgroups. Our multivariable analysis confirming zinc deficiency as an independent predictor of AKI, with a risk comparable to established risk factors such as heart failure and anemia (Table 5), positions zinc status among the modifiable clinical parameters that warrant routine attention. The identification of malnutrition as the strongest predictor underscores the complex interplay of nutritional deficiencies in CKD, whereas the independent effect of zinc deficiency highlights its distinct biological role beyond overall nutritional status.

In this study, AKI was identified using ICD diagnosis codes available within the TriNetX electronic health record database. Although this approach allows for large-scale analyses across diverse institutions, ICD coding is inherently less precise than the Kidney Disease: Improving Global Outcomes (KDIGO) definition, which requires serial creatinine measurements and urine output data. Reliance on administrative coding may therefore introduce misclassification, particularly in cases of subclinical or transient AKI that are not coded, or in situations where coding practices vary among healthcare organizations. Future studies incorporating laboratory-based definitions would provide greater diagnostic accuracy and external validity.

This study has several limitations that merit consideration when interpreting our findings. First, the observational design precludes definitive causal inferences, although the temporal relationship between baseline zinc measurements and subsequent outcomes provides stronger evidence than cross-sectional studies. Second, a key limitation is that serum zinc levels may be confounded by acute inflammation, infection, and albumin status, potentially resulting in misclassification of zinc deficiency. Although we included baseline albumin data and adjusted for albumin in our analyses, the absence of inflammatory markers such as CRP precludes more nuanced stratified analyses, and single-point zinc measurements may not fully capture chronic zinc status. Third, while our propensity score matching addressed measured confounders, unmeasured factors, such as dietary patterns, gastrointestinal absorption capacity, or genetic polymorphisms affecting zinc metabolism, could influence the observed associations. Fourth, the predominantly US-based healthcare systems in the TriNetX network may limit the generalizability to other healthcare settings or populations with different baseline zinc statuses or CKD management practices. Finally, the absence of information regarding zinc supplementation timing and adherence prevents the assessment of whether zinc repletion could modify the observed risk relationships.

## 5 Conclusion

Our retrospective analysis established zinc deficiency as a significant and independent risk factor for adverse outcomes in patients with CKD, with particularly pronounced effects on AKI development and mortality risk. The rapid manifestation of these effects, their persistence after excluding malnourished patients, and their consistency across diverse patient subgroups provide strong support for the direct, zinc-specific biological mechanisms underlying these associations. These findings suggest that routine zinc status assessments and targeted supplementation strategies warrant serious consideration in CKD management protocols. The degree of risk elevation linked to zinc deficiency is comparable to that of well-established major risk factors (e.g., heart failure), highlighting zinc status as a potentially significant but often overlooked contributor to clinical outcomes. Future research should focus on prospective interventional studies examining whether zinc supplementation can modify these adverse outcomes and establish optimal zinc repletion strategies for patients with CKD.

## Data Availability

The raw data supporting the conclusions of this article will be made available by the authors, without undue reservation.
